# Supplementary material for: Structure–function coupling and decoupling during movie watching and resting state: Novel insights bridging EEG and structural imaging
Source: Imaging Neurosci (Camb). 2025 Jan 23;3:imag_a_00448. doi: 10.1162/imag_a_00448 (PMC12319947; doi:10.1162/imag_a_00448)
Supplement: Supplementary Material [file imag_a_00448-supp.pdf]

Supplementary material

1 Alternative strategy for computing cut-off frequency adapted to EEG demonstrates tangible way to compute SDI

The cut-off frequency that characterizes low-frequency and high-frequency Eigenmodes was C = 2 across task conditions. Lower Eigenmodes carrying high power may be attributed to the spatial smoothness introduced by the source localization algorithm. Previous studies such as (Preti & Van De Ville, 2019) analyzing the structure-function association of fMRI signals preserved the smoothest Eigenmode to define the cut-off frequency. Previous EEG studies using different family of source localization algorithms revealed that the first connectome harmonics expresses the cortical activity disproportionately (Glomb et al., 2020; Rué-Queralt et al., 2021). Thus, we re-evaluated the choice of the cut-off by considering a different criterion, that only considers the graph spectrum for the non-constant eigenvectors.

We computed the SDI for the raw EEG signal following this strategy. The group-level Structural-Decoupling Index (SDI) results for the raw EEG signal (1-62.5 Hz) are presented in Fig 1. The median cut-off is shifted, C = 14 for Video 1, C = 13 for Rest, and C = 14 for Video 2 (compared to C = 2 earlier). A detailed breakdown of the new results and the comparison with the results in the manuscript are stated below.

Table 1: Differences in Coupling as a function of Task conditions and cut-off frequencies. ($C = 2$: Manuscript)

**Video 1**		**Rest**		**Video 2**	
$C = 14$	$C = 2$	$C = 13$	$C = 2$	$C = 14$	$C = 2$
Primary somatosensory cortex (Brodmann Area 1, SDI = -2.24)	Primary Somatosensory cortex (Area 2; SDI: -2.24)	Primary Motor Cortex (SDI = -2.20)	Primary somatosensory cortex (BA1, SDI: -1.78)	Primary somatosensory cortex (Area 2; SDI: -2.2)	Primary somatosensory cortex (Brodmann Area 1, SDI = -1.79)
Ventral anterior cingulate (SDI = -2.22)	Primary Sensory cortex (SDI: -1.8)	Primary somatosensory cortex (Brodmann Area 1, SDI = -2.24)	Visual V2 (SDI: -1.74)	Ventral anterior cingulate (SDI: -2.22)	Visual V3 (SDI: -1.77)
Brodmann area 47 in the frontal region SDI = -2.18	Second Visual area (V2; SDI: -1.8)	Third Visual Area (SDI: -2.12)	Visual V3 (SDI: -1.74)	Primary Motor Cortex (SDI: -2.18)	Visual V2 (SDI: -1.77)

Overall, the revised criterion has resulted in changes in SDI results, particularly in the cut-off values and spatial patterns of coupling and decoupling across different brain regions. Then, we sought to see the coherence of

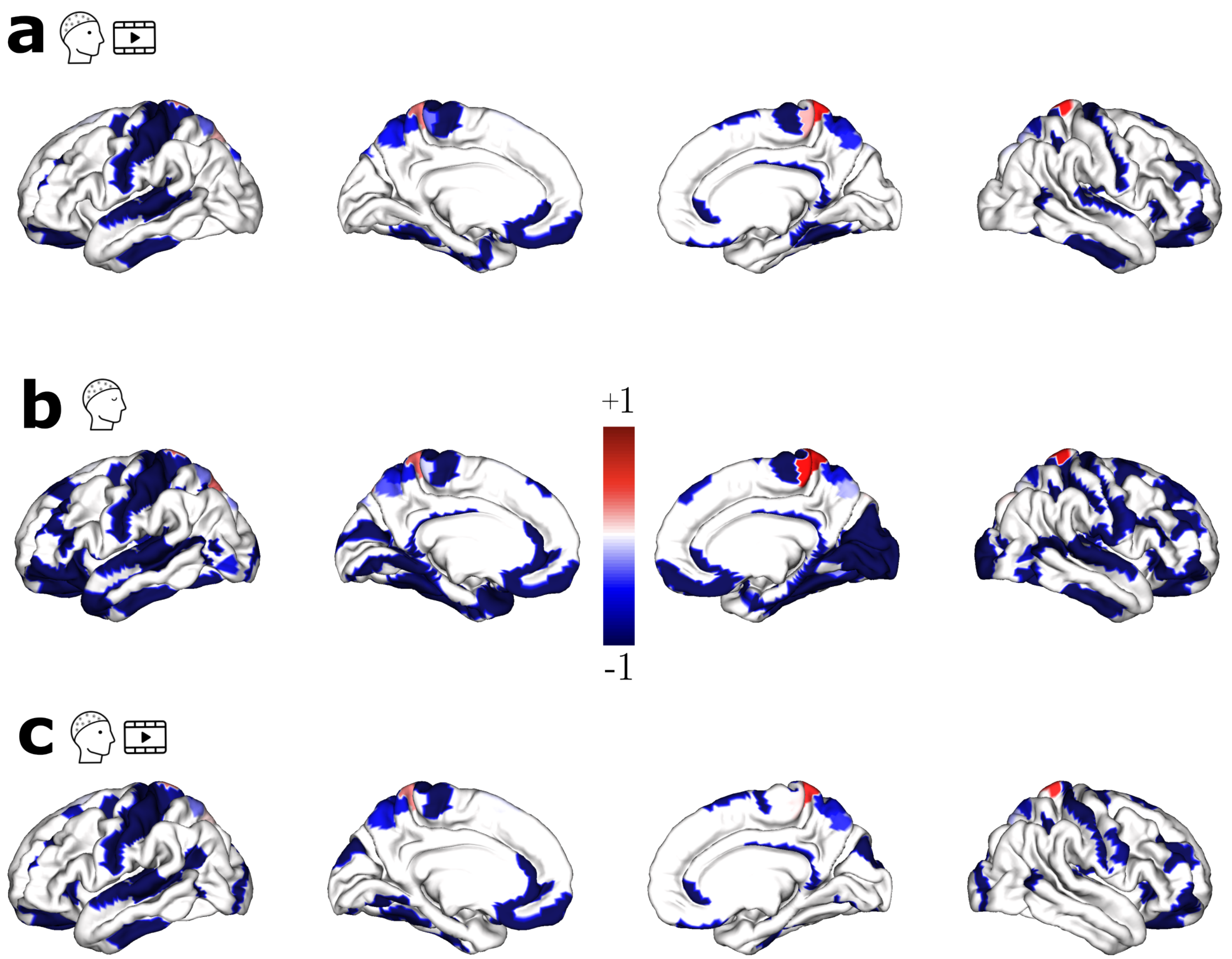

Figure 1: Group level SDI maps with the cut-off frequency chosen with a different criterion (i.e. disregarding the first constant eigenvector and choosing the frequency that dichotomizes the power spectrum of non-constant eigenvectors) (**a** Video1, **b** Rest, **c** Video2)

Table 2: Differences in Decoupling as a function of Task conditions and cut-off frequencies. ($C = 2$: Manuscript)

Video 1		Rest		Video 2	
$C = 14$	$C = 2$	$C = 13$	$C = 12$	$C = 14$	$C = 2$
Parahippocampal area 2 (SDI = 0.64)	Parahippocampal Area 2 (SDI: 0.62)	Parahippocampal area 2 (SDI = 0.61)	Parahippocampal area 2 (SDI: 0.71)	Posterior parietal cortex (SDI: 0.39)	Inferior Frontal Sulcal area (IFJp; SDI: 0.62)
Posterior parietal cortex (Area 5L; SDI: 0.38)	Inferior Frontal Sulcal area (IFJp; SDI: 0.5)	Posterior parietal cortex (Area 5L; SDI: 0.5)	Inferior Frontal Sulcal area (IFJp; SDI: 0.6)	Area 5m of Superior parietal lobule (SDI: 0.06)	Parahippocampal Area 2 (SDI: 0.49)
Area 5m of Superior parietal lobule (SDI: 0.17)	Posterior parietal cortex (Area 5L; SDI: 0.2)	Area 5m of Superior parietal lobule (SDI: 0.49)	Subgenual Anterior Cingulate cortex (Area s32; SDI: 0.38)	Lateral Area 7P of Superior parietal lobule (SDI: 0.07)	Subgenual Anterior Cingulate cortex (Area s32; SDI: 0.26)

Table 3: Contrast map between task conditions using different cut-off frequencies ($C = 2$: Manuscript)

Video 1 vs Rest		Video 2 vs Rest	
$C = 14$	$C = 2$	$C = 14$	$C = 2$
Area 5m of Superior parietal lobule (t(42) = -4.65)	Visual area 7 (V7; t(42) = -5)	Visual area V6A (t(42) = -5.5)	Visual areas (V3A, V6A; t(42) = -5.4, -5.4 respectively)
Visual area V6A (t(42) = -4.5)	Inferior parietal cortex (Area PGP; t(42) = -4.65)	Area 5m of Superior parietal lobule (t(42) = -4.5)	Area 5m of Superior parietal lobule (t(42) = -5.2)

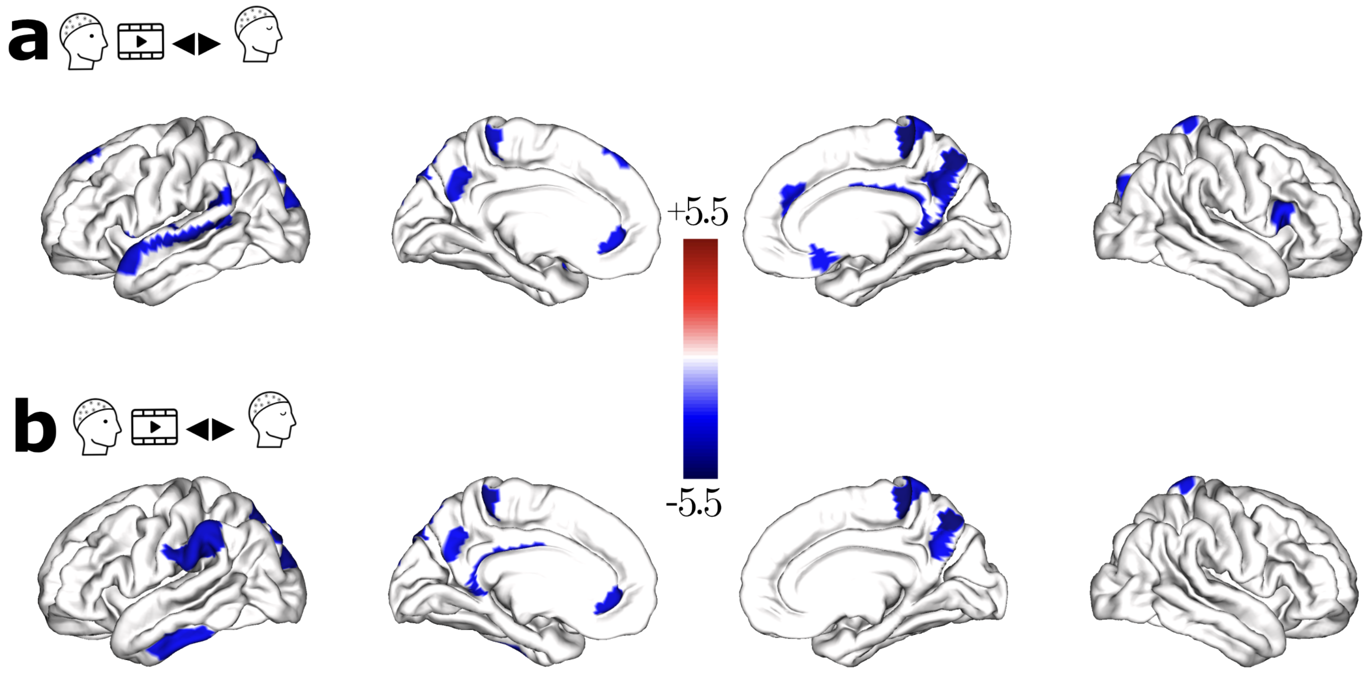

Figure 2: Contrast map between task conditions using the new cutoff strategy (**a** Video1 vs Rest, **b** Video2 vs Rest)

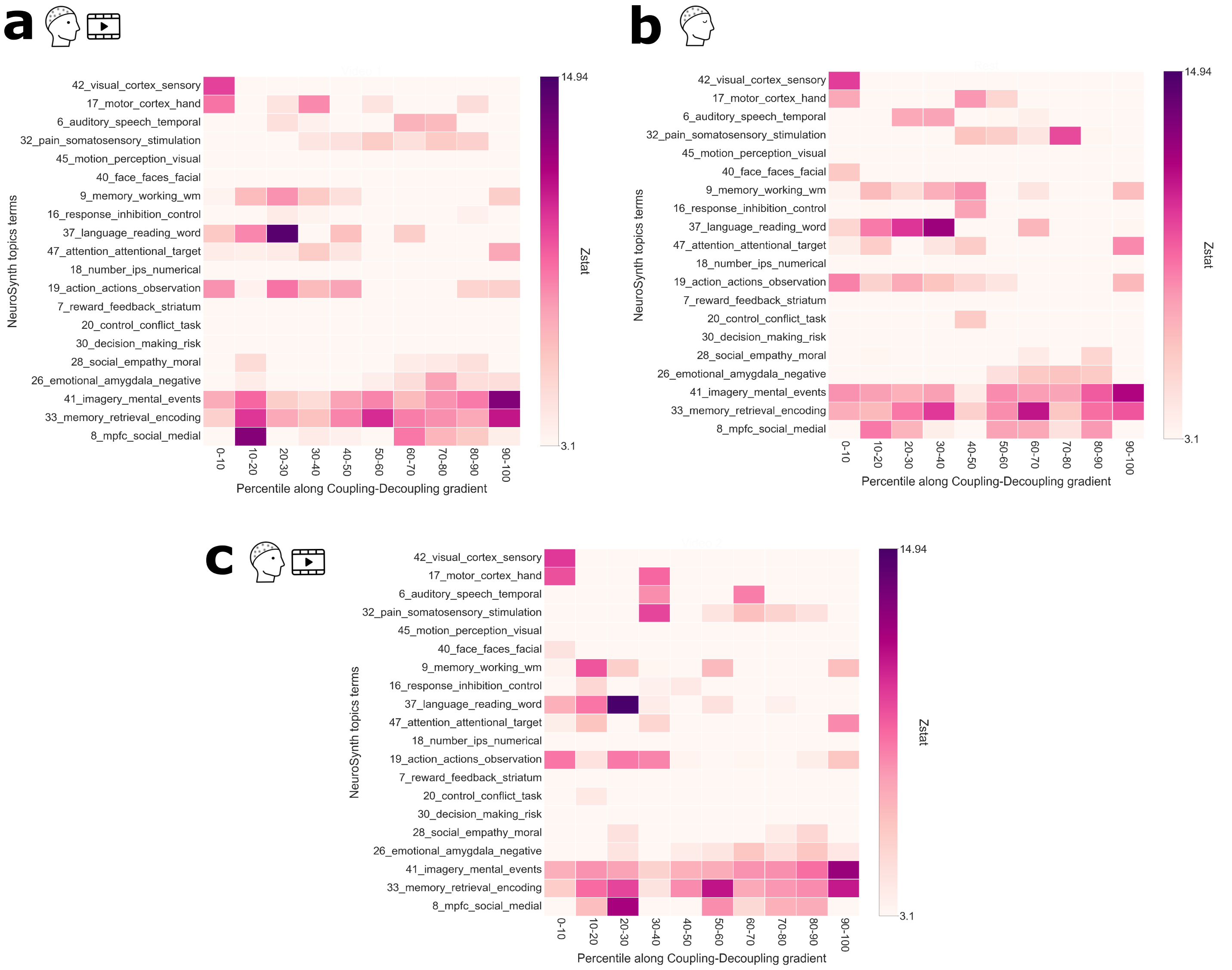

Figure 3: Decoding the spatial maps for different task conditions (**a** Video 1, **b** Rest, **c** Video 2) for which the SDI cutoff frequency is computed, excluding the constant eigenvector.

adapting the SDI computation for EEG by performing Neurosynth/Nimare decoding as well (Fig 3). These results can be interpreted as follows: sensory systems are associated with coupling, while the higher-order systems tend to run across the coupling-decoupling gradient. Albeit differences (higher order systems have high Zscore on the decoupling bin, as opposed to rather uniform spread out), our original interpretation still holds true.

These results after exclusion of the power of the constant eigenvector, and considering the whole power spectrum in the manuscript being complementary speak to the robustness of the methodology and the findings. The new setup could demonstrate a tangible way to approach the source-localized EEG signals.

2 Structure-Function coupling quantified for bandpass signals and Fourier coefficients reveals similar spatial distribution across frequency bands

We observed similar topography across frequency bands when the structure-function coupling is quantified for the envelope signal. One might wonder if applying Hilbert Transform obscures the subtle oscillations that cause this similarity. We investigated this by considering the bandpass signal, and fourier coefficients computed by Short-Term Fourier Transform (STFT).

We computed the SDI for the bandpass signal, and performed statistical thresholding with the same pipeline. Group-level maps are presented in Fig 4 for the various EEG frequency bands ranging from θ (4 - 8) to γ (30 - 40 Hz). We compared the spatial distribution across all pairs of frequency bands using group-level maps. Results reveal a high correlation with the average Spearman's rho of 0.84 across all pairs (SD: 0.02). This is comparable to the envelope signal (average rho being 0.86, SD being 0.02).

We analyzed the time and frequency characteristics of a source-localized EEG signal using the Short-time Fourier Transform (STFT). Specifically, we transformed the EEG data into time-frequency coefficients using a window length of 200 ms, a 50% overlap, and Hann windows. This window length was chosen as it offers a reasonable balance between the scale of frequencies (at least 5Hz) and the time samples (11 per second). These coefficients were then considered as the graph signal, and the structure-function coupling was quantified. When necessary, coefficients were averaged within the frequency range (e.g., two coefficients in the high-β range between 20 and 30 Hz). Group-level maps are presented in Fig 5 for the various EEG frequency bands ranging from θ (4 - 8) to γ (30 - 40 Hz). We computed the whole-brain spatial similarity across all pairs of frequency bands at the group level. Results reveal the average rho being 0.73 (SD: 0.13). Though there is a slight drop in averaged rho, the spatial similarity is nevertheless high.

Despite considering the bandpass signal, and STFT coefficients as graph signals, we still find comparable spatial similarity, indicating the oscillation that might be preserved is not sufficient for the SDI metric to show clear differences.

3 Eigenmodes of the consensus structural connectome

4 Decoding SDI maps with automatic ordering of topics highlight differences across task conditions

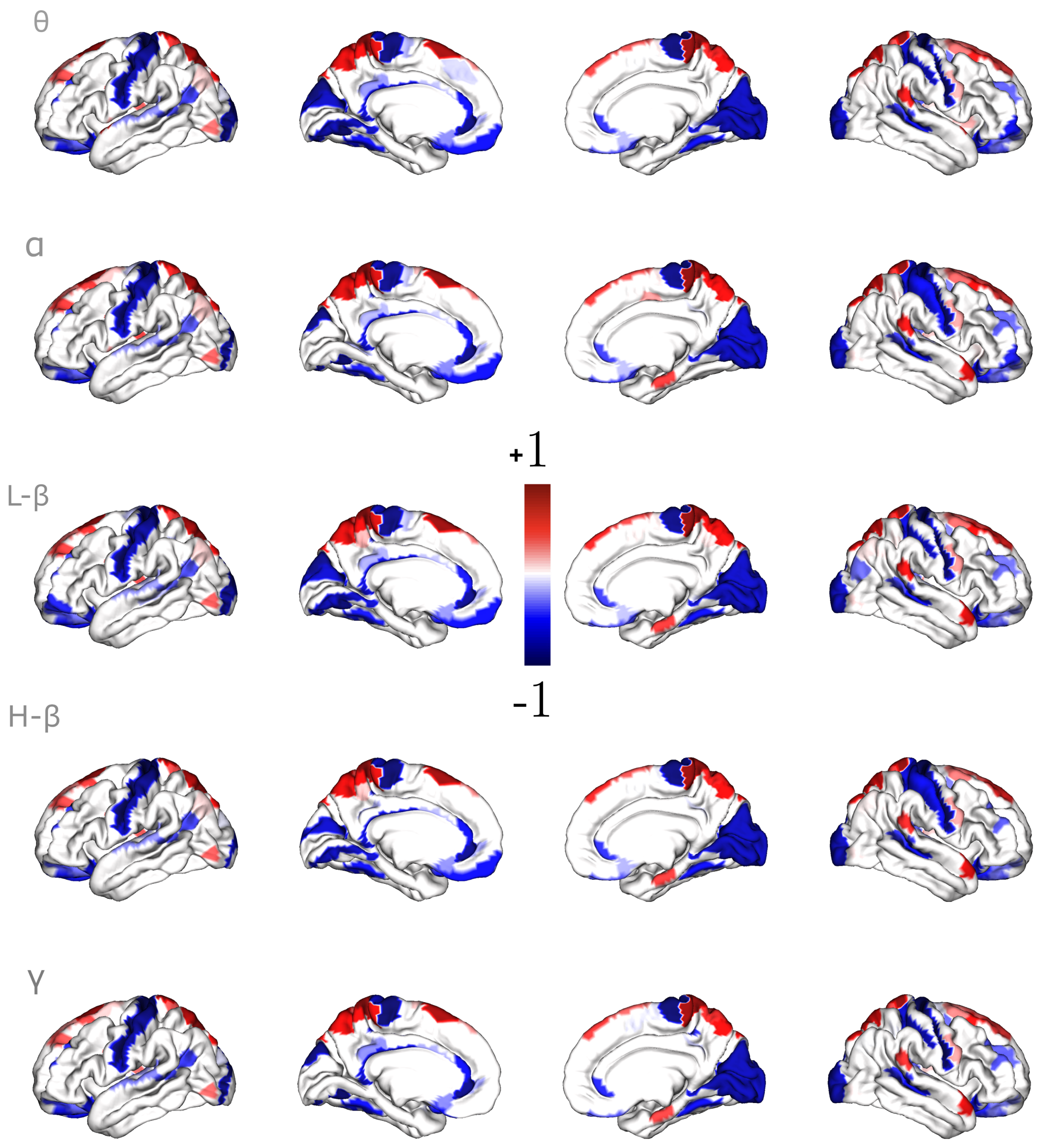

Figure 4: Group SDI maps of the link between bandpass filtered signals and the anatomy

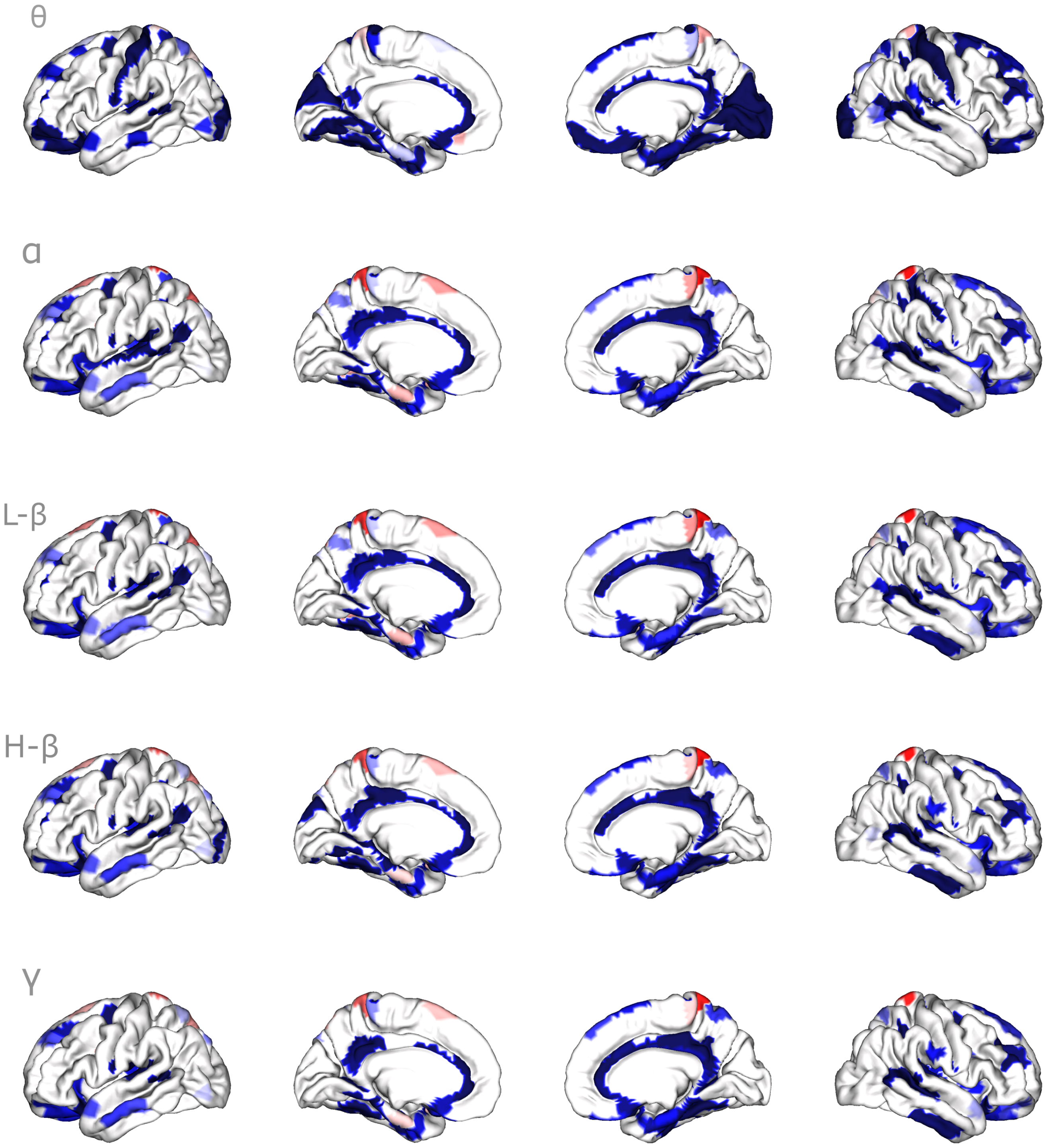

Figure 5: Group level SDI maps depicting the link between the STFT coefficients of the cortical signal and the anatomy.

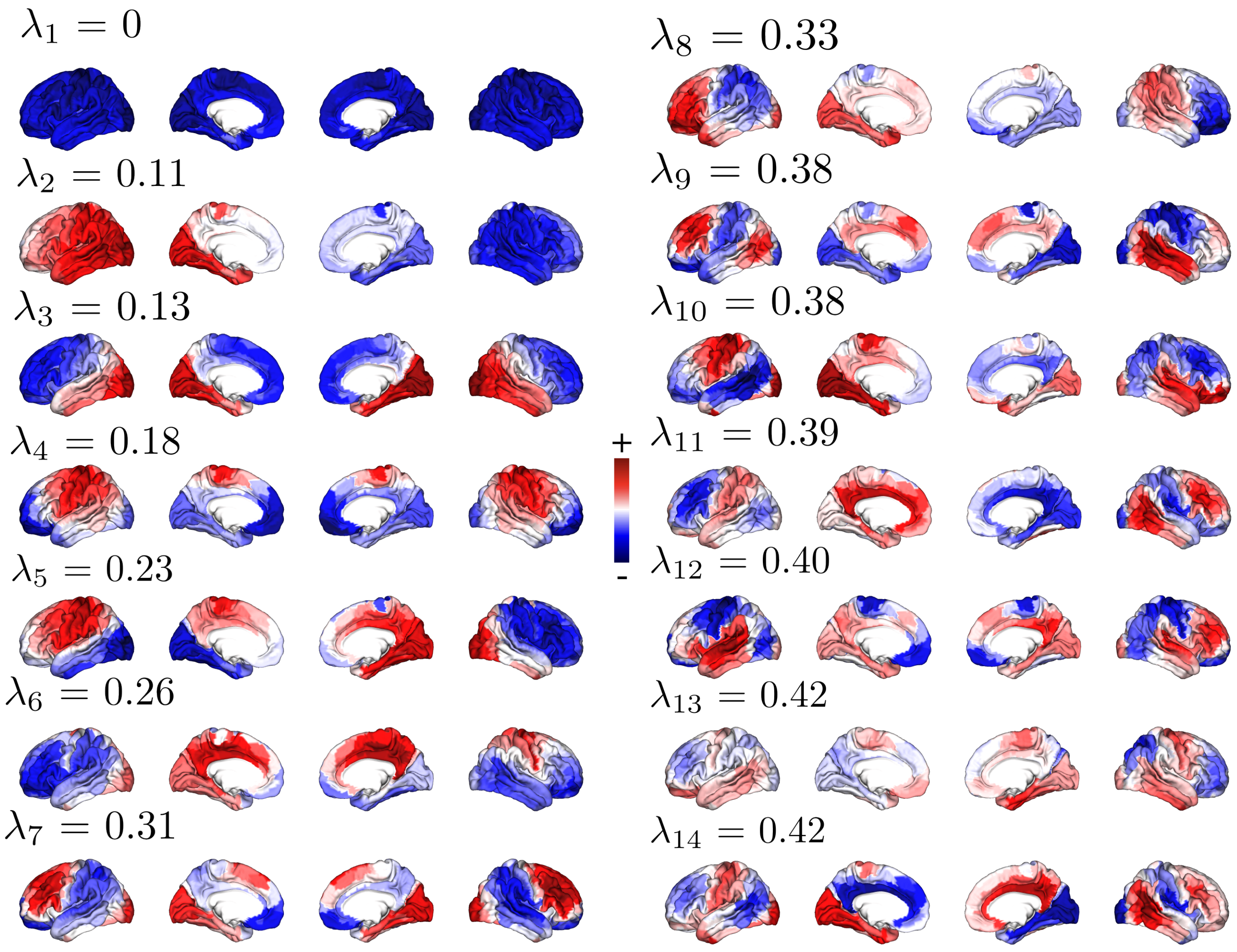

Figure 6: First 14 Eigenmodes of the laplacian of the group-averaged structural graph

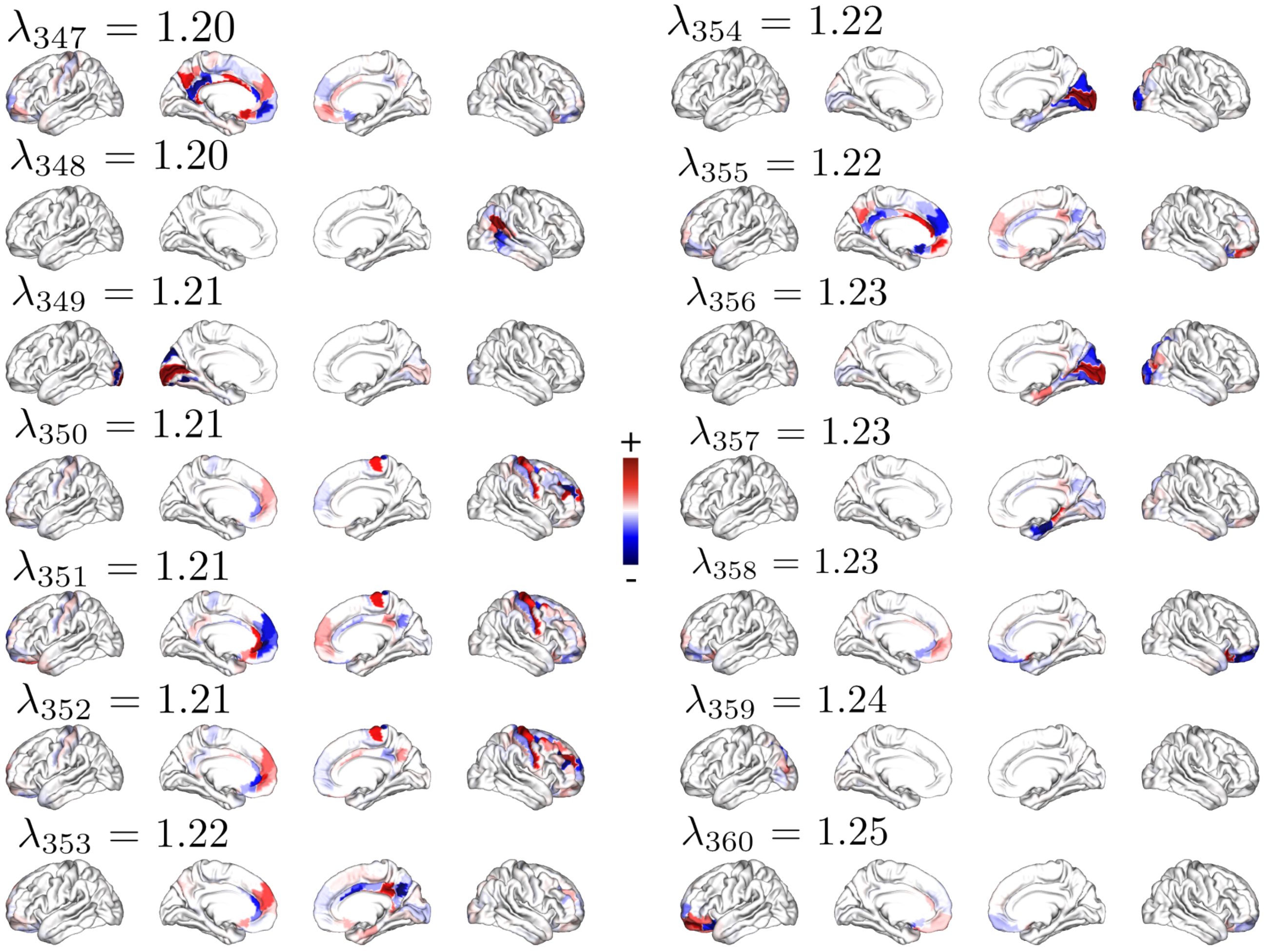

Figure 7: Last 14 Eigenmodes of the laplacian of the group-averaged structural graph

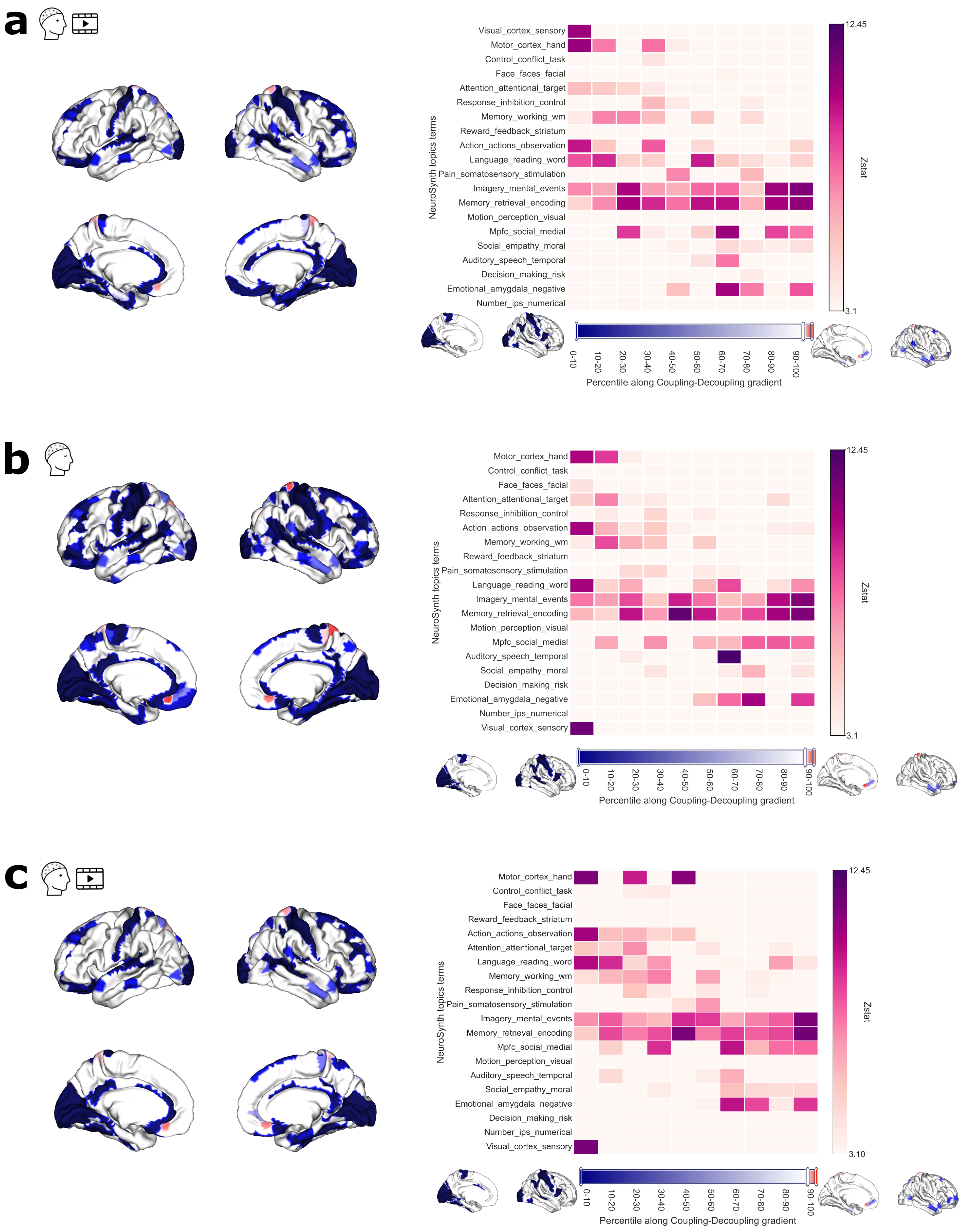

Figure 8: Decoding results with an automated ordering of the topics, as in previous studies
